# Transvers testicular ectopia: A case report and literature review

**DOI:** 10.1016/j.ijscr.2019.11.007

**Published:** 2019-11-12

**Authors:** Tural Abdullayev, Mevlit Korkmaz

**Affiliations:** a Department of Pediatric Surgery, Medical Park Gebze Hospital, Güzeller, Kavak Cd. No: 5, 41400 Gebze, Kocaeli, Turkey; b Department of Pediatric Surgery, EMSEY Hospital, Çamlık Mah, Selçuklu Cad. No: 22 Pendik, İstanbul, Turkey

**Keywords:** Transverse testicular ectopia, Trans septal orchiopexy, Undescended testis, Crossed testicular ectopia, Persistent mullerian duct syndrome

## Abstract

**İntroduction:**

Transverse testicular ectopia (TTE) is a rare anomaly characterized by the presence of both testicles in the same hemiscrotum or inguinal region. The most common clinical findings of TTE are unilateral nonpalpable testis in the scrotum and inguinal hernia on the side of palpable testis in the scrotum. It should be kept in mind that TTE may coexist with Persistent Mullerian Duct Syndrome (PMDS). Therefore, appropriate treatment should be performed considering PMDS.

**Presentation of case:**

Type 2 transverse testicular ectopia was diagnosed in one patient who was operated with bilateral undescended testis. He was treated with transseptal orchiopexy and excision of mullerian structures.

**Discussion:**

In case of TTE with PMDS, optimal surgical approach with orchiopexy and excision of Müllerian duct is necessary. Intraoperative aggressive dissection of vas deferens and testicular vessels should be avoided in TTE patients. They should be closely followed because of the increased risk of malignant transformation in the postoperative period.

**Conclusion:**

An investigation of transvers testicular ectopia should be performed in all nonpalpable undescended testis anomalies.

## Highlights

• TTE associated with PMDS is a rare case which is incidentally discovered during surgery of undescended testis.
• Early diagnosis and treatment is necessary to prevent malignancy.
• Follow-up for fertility assessment in the latter years should be counselled.

## 1 Introduction

TTE is a rare anomaly characterized by the presence of both testicles in the same hemiscrotum or inguinal region. It is usually found incidentally in patients operated for inguinal hernia or undescended testicles. Preoperative ultrasonography and laparoscopic evaluations are helpful in diagnosis, and the treatment of TTE. Standard treatment of TTE is mainly surgery, including inguinal hernia repair, transseptal orchiopexy, and the repair of congenital anomalies [1]. In this case study, we report a case of type 2 transvers testicular ectopia. The work has been reported in line with the SCARE criteria [2]. Written informed consent was obtained from the legally authorized representative(s) for anonymized patient information to be published in this article.

## 2 Presentation of the case

**Fig. 1:**
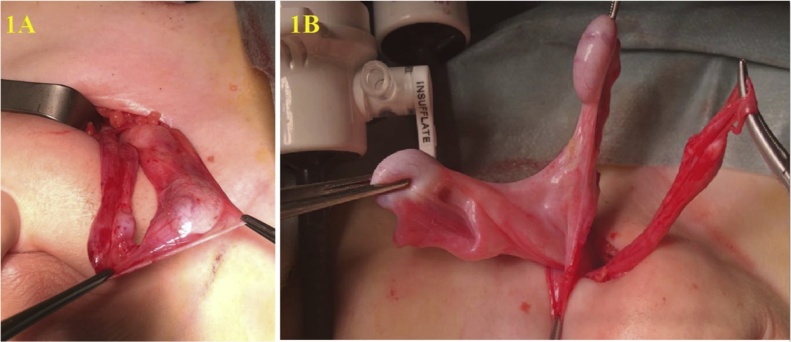
**A.** Common Processus Vaginalis **B.** Testicles and Persistent Mullerian Structures.

**Fig. 2:**
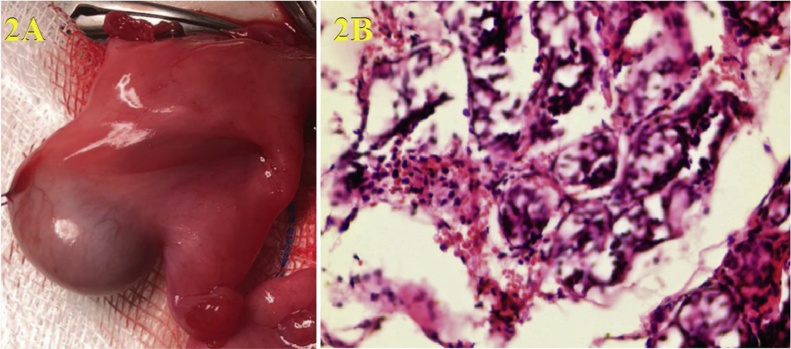
**A.** Testicle, fallopian tube, and its fimbriae **B.** The biopsy sample of infantile testis tissue.

**Fig. 3:**
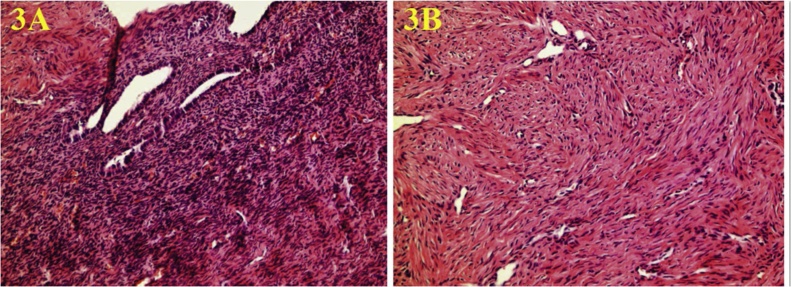
Infantile uterus **A.** Endometrium **B.** Myometrium.

**Fig. 4:**
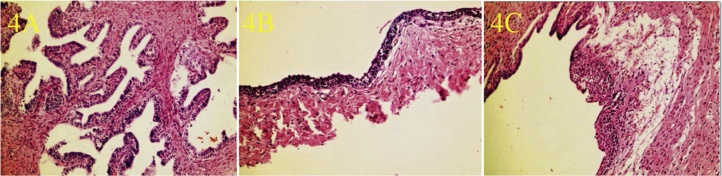
Histopathological appearance of the fallopian tube (**A**), the cervix (**B**), and the vagina (**C**).

An eight-month-old male patient was admitted to our hospital, pediatric surgery clinic with the complaint of bilateral undescended testes. According to medical history; he was born at term, and there was no medical problem during the postnatal period. On the physical examination, an uncircumcised penis, left undescended testis in the inguinal canal, and unpalpable right testis were observed. The urethral meatus was in normal localization, and there was no finding related to the hernia in both inguinal canals. In ultrasonography, right testicle was detected in the inguinal canal, but left testicle could not be detected. Hence, the patient underwent laparoscopy for diagnostic and therapeutic purposes:

The operation was started by entering with a 3-mm port from the umbilicus. In the diagnostic laparoscopy performed with 3-mm optics, the left testis was observed in the inguinal canal, and the right testis was close to the left inguinal canal opening. The right testis was ectopically located on the left side with the left testis. The dissection of the adherent hernia sac was decided during this operation in this patient with TTE (Fig. 1A). However, the common mesorchium of the two testicles was very thick, and this might result from the coexistence of nonregressed Mullerian structures and type 2 TTE (Fig. 1B). Bilateral orchiopexy was performed by preserving the bilateral vas deferens, and testicular vessels, removing the remnants of Mullerian structures (uterus, fallopian tubes, and fimbria-like structure). The left testis was placed and fixed on the ipsilateral pouch of the scrotum, and the right testis was pulled transseptal from the left side to the right hemiscrotum, and then fixed there. Biopsies from testis, uterus, and fallopian tubes confirmed that these structures were testicles, and Mullerian structures (Fig. 3, Fig. 4). Testicular biopsies showed infantile testicular tissue in the static phase (Fig. 2B ). Peripheral blood chromosome analysis of the patient revealed 46XY genotype.

## 3 Discussion

TTE may be seen alone or accompanied by inguinal hernia, hypospadias, scrotal anomalies, true hermaphroditism, and PMDS. The ectopic testis may be located in the abdomen, at the level of the internal inguinal ring or in the same hemiscrotum [1]. The incidence of this anomaly is one in 4 million in children [3]. It was first described by Von Lenhossek in 1886 in an autopsy performed by his father [4]. Approximately 260 cases have been reported by scientists [5].

TTE may occur with different clinical conditions. Both testicles have a common processus vaginalis sac, and inguinal hernia is the most common situation where the ectopic testis should be present. On the side of the ectopic testis, incarcerated inguinal hernia is an expected condition. Transverse testicular ectopia is the first pathology to be considered in the presence of unilateral non-palpable testis and contralateral reducible or irreducible hernia [6]. In the classification of Gauderer et al., TTE is divided into three types according to clinical findings [1]. Type 1 is the most common form of TTE (40–50%). In some studies, the coexistence of TTE has been reported in patients presenting with the incarcerated inguinal hernia. Thus, it should be considered that type 1 TTE may be present in cases with the unilateral incarcerated hernia, and contralateral nonpalpable undescended testis [7]. Type 2 TTE is the second most common form (30 %) and is associated with PMDS. The coexistence of PMDS with TTE was first described by Jordan in 1895 [4]. PMDS is characterized by the presence of Mullerian residues (fallopian tube, 1/3 of uterus and vagina) in male phenotype. It is a disorder of male sex development (DSD) caused by Mullerian Inhibitory Factor (MIF) deficiency secreted from the fetal Sertoli cells (46 XY DSD). The causative gene of MIF is located on the short arm of chromosome 19, and it usually has an autosomal recessive pattern, but it sometimes reveals X-linked recessive inheritance [8]. In some cases, the problem is at the MIF receptor level and PMKS develops as a result of disruption of activation of MIF in target organs. In the AMHR2 gene analysis, it was reported that homozygous c.24 G > A (p.W8X) mutation may cause AMH receptor resistance and cause type 2 TTE [9]. PMDS is usually seen in three forms. Type 1 Male form PMDS seems as partially descended testis, and hernia uteri inguinalis. Type2 Male form with transverse testicular ectopia. Type 3 Female form PMDS is characterized by bilateral intraabdominal testes and Mullerian structures at the ovarian position. In all three forms, the subject has the male phenotype and 46 XY genotype. Azoospermia is prevalent due to intrinsic problem in the testes [10]. The effect of PMDS on the embryological development of TTE may be explained by mechanical effects. Persistent Mullerian duct may interfere with the expected descent of the testicles mechanically or it may push both testicles towards the same hemiscrotum, and cause the development of TTE [11]. The development of malignancy after puberty has been reported in previous studies. Close follow-up of the patients is recommended because the low fertility rate and increased malignancy risk are the primary outcomes of this anomaly. The cases of adenocarcinoma, cystadenocarcinoma, and squamous cell carcinoma caused by the residues of Mullerian duct were reported [13]. Type 3 TTE is the third most common (20 %) form and is associated with anomalies (hypospadias, scrotal anomalies, fused vas deferens, seminal vesicle cysts, testicular microlithiasis) other than PMDS. Fused vas deferens is a rare pathology associated with TTE, but it may prevent the descent of testis into the scrotum [14]. Rarely, TTE might be associated with a blindly ending vas deferens anomaly [15].

Ultrasonography, MRI, and laparoscopy may be used for diagnosis in addition to physical examination [16].

The basic treatment principles of TTE are the preservation of fertility, the repair of congenital anomalies, and hernia, orchiopexy, follow-up due to increased malignancy risk. Surgery may be performed with inguinal approach, laparoscopy, laparoscopy assisted inguinal aproach, or laparotomy. Laparoscopic exploration will improve the appearance of the anatomy (Mullerian structures, common vas deferens, etc.).

The surgical techniques used in the treatment of TTE are transseptal orchiopexy and transperitoneal orchiopexy. In the transseptal orchiopexy method, the ectopic testis is moved from the window created in the scrotal septum to the opposite side. In the transperitoneal orchiopexy method, the ectopic testis is placed to the extraperitoneal area by crossing the root of the penis and fixed into the other side of the hemiscrotum [17]. However, vas deferens and testicular vessels must be long enough to use this technique. The preferred surgical treatment modality for TTE is the transseptal orchiopexy technique (Ombredanne’s technique). The treatment algorithm of this technique was developed by Bascuna et al. according to the length of the funicular elements [18], and this algorithm was modified by Raj et al. [19]. Unlike Bascuna et al., Raj et al. argued that extensive dissection should not be performed in order to understand the anatomical associations between vas deferens and testicular vessels in this algorithm. In addition, they suggested fixing both testicles into the same hemiscrotum in cases where transseptal orchiopexy was not feasible. Contralateral transseptal orchiopexy method should be applied in cases where funicular structures (vas deferens, testicular vessels) are short, and transseptal orchiopexy is not suitable for the ectopic testis (Modified Ombradanne Operation). For example, right-sided ectopic testis with short funicular elements should be placed into the right hemiscrotum. However, it should be placed into the left hemiscrotum through the hole in the scrotal septum if this ectopic testis has long funicular elements [20]. If the funicular structures of both testicles are short, and none of the transseptal or contralateral transseptal orchiopexy techniques cannot be applied, both testicles can be placed into the same hemiscrotum [19].

The primary surgical approach in type 2 TTE cases with PMDS is similar to type 1 TTE. Besides, the excision of Mullerian structures is needed in type 2 TTE cases. The main purpose of the surgery is not to damage the vas deferens, which is usually located lateral to the uterus and testicular vessels. Microsurgical techniques can be performed to separate fused structures. But, in spite of these techniques, if the fusion is at an advanced level and the injury to the vas deferens is unavoidable, salpingectomy should be performed at the proximal level, the pedicle of the myometrium should be left intact, and the mesentery of the fallopian structure attached to the upper pole of the testis should be left without separation from the testicle due to the injury risk of the epididymis [13]. Patients with residual Mullerian tissue should be closely followed due to the increased risk of malignant transformation in the long term.

## 4 Conclusion

In cases with the undescended testis, it should be considered that the underlying pathology may be TTE. On physical examination, the inguinal canal on the side of the descended testis into the scrotum should be palpated for the presence of the ectopic testis up to the internal inguinal ring, if there is a suspicion for TTE.

## Sources of funding

The case report had no sponsors.

## Ethical approval

This case report is exempt from ethical approval by our institution

## Consent

Written informed consent was obtained from the patient’s relatives for publication of this case report and accompanying images. A copy of the written consent is available for review by the Editor-in-Chief of this journal on request.

## Registration of research studies

This is not a ‘first in humans’ report, so it is not in need of registration.

## Guarantor

Mevlit Korkmaz, MD, Assoc. Prof2 Email: mevlitkorkmaz@gmail.com.

**Address:** Çamlık Mah. Selçuklu Cad. No:22 Pendik İstanbul Türkiye.

**OrcID:** https://orcid.org/0000-0001-5494-5868.

## Provenance and peer review

Not commissioned, externally peer-reviewed.

## CRediT authorship contribution statement

**Tural Abdullayev:** Writing - original draft, Writing - review & editing, Data curation, Conceptualization. **Mevlit Korkmaz:** Writing - original draft, Writing - review & editing, Data curation.

## Declaration of Competing Interest

No potential conflicts of interest.

## References

[1] Gauderer M.W., Grisoni E.R., Stellato T.A., Ponsky J.L., Izant R.J. (1982). Transverse testicular ectopia. J. Pediatr. Surg..

[2] Agha R.A., Borrelli M.R., Farwana R., Koshy K., Fowler A., Orgill D.P., For the SCARE Group (2018). The SCARE 2018 statement: updating consensus surgical case report (SCARE) guidelines. Int. J. Surg..

[3] Esteves E., Pinus J., Maranhao R.F., Abib Sde C., Asis S.V., Pinus J. (1995). Crossed testicular ectopia. Sao Paulo Med. J..

[4] Aditi S. (2015). Agrawalcorresponding author and Raman Kataria. Persistent Mullerian Duct Syndrome (PMDS): a Rare Anomaly the General Surgeon Must Know About. Indian J. Surg..

[5] Rajesh A., Farooq M. (2017). A rare case of male pseudohermaphroditism-persistent Mullerian duct syndrome with transverse testicular ectopia - case report and review of literature. Int. J. Surg. Case Rep..

[6] Boyle Thomas A., Perez Eduardo A., Diez Ricardo, Sola Juan E., Sanz Eva E., Garcia Ana, Fuentes Ennio J. (2019). Transverse testicular ectopia discovered following reduction of an inguinal hernia. J. Pediatr. Surg..

[7] Gujar N.N.1, Choudhari R.K., Choudhari G.R., Bagali N.M., Mane H.S., Awati J.S., Balachandran V. (2011). Male form of persistent Mullerian duct syndrome type I (hernia uteri inguinalis) presenting as an obstructed inguinal hernia: a case report. J. Med. Case Rep..

[8] Ren X.1, Wu D.1, Gong C. (2017). Persistent Mullerian duct syndrome: a case report and review. Exp. Ther. Med..

[9] Korkmaz Özlem, Özen Samim, Özcan Nurhan, Bayındır Petek, Şen Sait, Onay Hüseyin, Gökşen Damla, Avanoğlu Ali, Özkınay Ferda, Darcan Şükran (2017). Persistent mullerian duct syndrome with transverse testicular ectopia: a novel anti-mullerian hormone receptor mutation. J. Clin. Res. Pediatr. Endocrinol..

[10] Solanki S., Gowrishankar Jadhav V., Babu M.N., Ramesh S. (2015). Female form of persistent Mullerian duct syndrome: rare entity. Urol. Ann..

[11] Karnak I., Tanyel F.C., Akçören Z., Hiçsönmez A. (1997). Transverse testicular ectopia with persistent Mullerian duct syndrome. J. Pediatr. Surg..

[13] Farikullah J., Ehtisham S., Nappo S., Patel L., Hennayake S. (2012). Persistent Mullerian duct syndrome: lessons learned from managing a series of eight patients over a 10-year period and review of literature regarding malignant risk from the Mullerian remnants. BJU Int..

[14] Chacko J.K., Furness P.D., Mingin G.C. (2006). Presentation of fused vas deferens. Urology.

[15] Dhua A.K.1, Varshney A.1, Bhatnagar V. (2016). Transverse testicular ectopia with a blind ending vas deferens. Indian J. Urol..

[16] Lam W.W., Le S.D., Chan K.L., Chan F.L., Tam P.K. (2002). Transverse testicular ectopia detected by MR imaging and MR venography. Pediatr. Radiol..

[17] Bothra J.M., Shah H.S., Jayaswal S., Sandlas G. (2014). Transverse testicular ectopia: a rare anomaly. J. Pediatr. Neonatal Care.

[18] Bascuna R., Ha J., Lee Y., Lee H., Im Y., Han S. (2015). Transverse testis ectopia:diagnostic and management algorithm. Int. J. Urol..

[19] Raj Vinod, Redkar Rajeev, Krishna Swathi, Tewari Shruti (2017). Rare case of transverse testicular ectopia – case report and review of literature. Int. J. Surg. Case Rep..

[20] Divarcı E., Ulman I., Avanoglu A. (2011). Transverse testicular ectopia treated by transseptal contralateral transposition: case report. Eur. J. Pediatr. Surg..

